# Fabrication and Characterization Techniques of In Vitro 3D Tissue Models

**DOI:** 10.3390/ijms24031912

**Published:** 2023-01-18

**Authors:** Rohin Shyam, L. Vinod Kumar Reddy, Arunkumar Palaniappan

**Affiliations:** 1 School of Bio Sciences and Technology (SBST), Vellore Institute of Technology (VIT), Vellore 632014, Tamil Nadu, India; 2 Centre for Biomaterials, Cellular, and Molecular Theranostics (CBCMT), Vellore Institute of Technology (VIT), Vellore 632014, Tamil Nadu, India; 3 Department of Biomedical Engineering, University of North Texas, Denton, TX 76207, USA

**Keywords:** in vitro models, 2D and 3D cell cultures, 3D tissue models, 3D bioprinting, confocal microscopy

## Abstract

The culturing of cells in the laboratory under controlled conditions has always been crucial for the advancement of scientific research. Cell-based assays have played an important role in providing simple, fast, accurate, and cost-effective methods in drug discovery, disease modeling, and tissue engineering while mitigating reliance on cost-intensive and ethically challenging animal studies. The techniques involved in culturing cells are critical as results are based on cellular response to drugs, cellular cues, external stimuli, and human physiology. In order to establish in vitro cultures, cells are either isolated from normal or diseased tissue and allowed to grow in two or three dimensions. Two-dimensional (2D) cell culture methods involve the proliferation of cells on flat rigid surfaces resulting in a monolayer culture, while in three-dimensional (3D) cell cultures, the additional dimension provides a more accurate representation of the tissue milieu. In this review, we discuss the various methods involved in the development of 3D cell culture systems emphasizing the differences between 2D and 3D systems and methods involved in the recapitulation of the organ-specific 3D microenvironment. In addition, we discuss the latest developments in 3D tissue model fabrication techniques, microfluidics-based organ-on-a-chip, and imaging as a characterization technique for 3D tissue models.

## 1. Introduction

In vitro two-dimensional (2D) cell culture methods are a widely used tool for understanding biological functions such as cellular interaction, mechanisms of disease initiation and progression, production of proteins, cellular biology, and, more recently, the development of engineered tissue mimics. In a 2D environment, cells are grown as a monolayer over a flat plastic surface, where they adhere and spread. However, the simplicity of this model makes the depiction and simulation of complex tissue structures challenging. Two-dimensional monolayer cultures have been used for decades to study the cellular responses to biochemical and biophysical cues. These systems do not always mimic human physiological conditions despite providing significant advancements in the understanding of cellular behavior [1], thereby resulting in non-predictive results.

In recent years, the paradigm has shifted towards three-dimensional (3D) cell cultures. Increasing research-based evidence suggests that 3D tissue models are a better option for mimicking complex tissue or organ architecture (cell–cell and cell–matrix interactions) and physiology [2]. These models are gaining importance from basic research to advanced application-based research such as drug testing/screening and other translational purposes. In human tissue, cells are encapsulated within extracellular matrix (ECM) proteins in a 3D environment [3]. The ECM function under defined biophysical and biochemical signals, which regulate cellular functions such as proliferation, adhesion, migration, differentiation, and morphogenesis and maintain homeostasis [4]. Hence, different 3D models are evolving with the combination of cells and proteins to recapitulate native organs and the cellular microenvironment. This aids in understanding the various organs and tissue functions under a controlled laboratory setting and offer the possibility to generate organ-specific and personalized drug testing platforms [5].

Recent advances in microfabrication techniques and tissue engineering technology have influenced the development of complex culture systems and biomimetic microfluidic platforms to capture the structural and functional complexity of the native physiological environment. Tissue engineering is a subfield of regenerative medicine that aims to repair, replace, or regenerate tissues or organs. This is achieved through the translation of fundamental principles of physics, chemistry, and biology combined with the principles of materials engineering and cell transplantation. The goal of this approach is to mimic native tissues that can function as medical devices with therapeutic benefits to regenerate damaged tissues, function as a platform to study drug cytotoxicity at a cellular and molecular level, and model disease under laboratory conditions [6,7]. With these advancements, 3D models with ECM-mimicking proteins could recapitulate the microarchitecture and functional cellular environment of the native organ. In recent years, organ-on-the-chip technology has been gaining prominence due to its ability to simulate organ-level physiology by recreating the multicellular connections and interfaces, vascular perfusion, mechanical cues, and chemical gradient under highly controlled environments.

**Figure 1:**
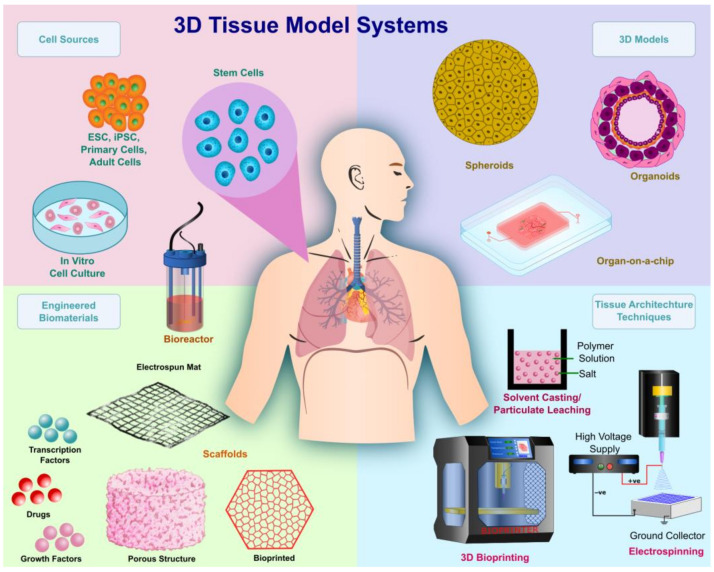
Schematic representation of considerations in the fabrication of 3D tissue models.

The bioengineering and designing of complex biomimetic tissue for model systems involve considering several design characteristics and parameters. A 3D tissue model system can be generated through the fabrication of spheroids and organoids; however, while being able to provide a 3D microenvironment, a critical challenge with these systems is the lack of vasculature, which is essential in providing oxygen and nutrients while removing metabolic waste from cells. Alternatively, a scaffold that mimics the ECM is generated via techniques such as 3D bioprinting, electrospinning, and solvent casting/particulate leaching (SCPL) to create porous structures that house the cells, growth factors, vasculature, and transcription factors. The choice of biomaterial to generate the ECM is critical. There are a variety of natural and synthetic biomaterials available, with each having its own benefits and limitations. There has been an increased interest in the combination of biomaterials to generate hybrid biomaterials, which enhance the structural and biological properties of biomaterials. Another consideration is the choice of cells, which is dependent on the tissue being modeled. Stem cells are state-of-the-art in tissue engineering due to their differentiation potential into any cell lineage. Figure 1 summarizes the essential considerations in the realm of 3D tissue models. In the following sections, we discuss the types of 3D tissue models, types of biomaterials and their key characteristics, techniques used in the mimicking of tissue architectures and generation of porous scaffold structures, the types of cells used in 3D models, and their advantages and disadvantages, concluding with the imaging modalities of tissue architecture.

It is often challenging to directly study and observe the complex mechanisms of human development and disease due to a lack of experimental accessibility to biological processes. As a result, the use of model systems that recapitulate these functions ex vivo has been of primary interest to researchers. Two-dimensional culture systems have been established as standard protocols to observe cellular behavior; however, these systems do not completely recapitulate the cellular microenvironment. For instance, the epithelia of the small intestine is an active and rapidly renewing tissue that can undergo tissue widening and form compact folds, invaginations, evaginations, and wavy morphologies [8]. Similarly, in the cardiovascular system, myocardial fibrillar proteins form a 3D complex structure that changes orientation during systole and diastole, resulting in the cardiac tissue undergoing cyclic stress, torsion, and compression [9,10]. These microenvironments represent a major challenge to replicate in vitro. The replication of tissue-specific conditions within a 3D model can offer the ability to study these complex mechanisms and enable a deeper understanding of the role in human development and disease progression and as a platform for drug testing. The key differences between 2D and 3D cell cultures are tabulated in Table 1. There are several strategies to fabricate and characterize 3D tissue models. In this review, we have explored the various types of 3D model strategies involved in the fabrication of complex models. Importantly, we have also explained unique imaging techniques involved in the characterization of 3D tissue models.

## 2. Types of 3D Tissue Models

### 2.1. Anchorage Independent (Non-Scaffold Based) 3D Tissue Models

#### 2.1.1. Spheroids

**Figure 2:**
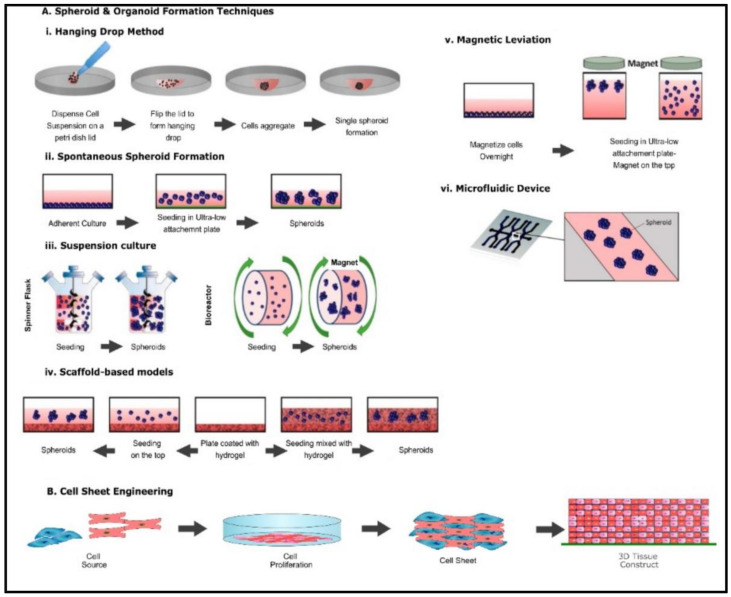
Common fabrication techniques used for the creation of spheroids, organoids, and cell sheet (**A**) (i) Hanging drop method (ii) Spontaneous spheroid formation (iii) suspension culture (iv) ECM method (v) Magnetic levitation method, (vi) Microfluidic device method. Altered and reproduced with permission from [27] under Open Access CC BY 4.0. MDPI (**B**) Schematic representation of cell sheet engineering.

Spheroids are perfectly spherical cellular aggregates in suspension generated from primary cell types and cell lines. The term was coined in 1970 by Sutherland et al. when the group dissociated Chinese Hamster V79 lung cells which formed spherical aggregates [21]. There are various techniques involved in the fabrication of spheroid, including the hanging drop technique, microwell hanging drop technique, liquid overlay technique, microwell array from micropatterned agarose wells, rotating wall vessel, and magnetic levitation (Figure 2A(i–vi)) [22,23]. Microfluidic technology and 3D bioprinting have also been utilized in the generation of spheroids [24,25,26]. The most common applications of spheroids are in creating tumor models, stem cell research, tissue engineering, and transplantation therapy. The key advantages of using this method as a 3D tissue model are that it facilitates cell–cell and cell–matrix interactions providing a physiochemical environment similar to in vivo while maintaining intrinsic phenotypic properties and improving the viability and proliferation of cells [23]. 

However, there are several drawbacks to this method. Due to the lack of vasculature within the aggregates, the supply of nutrients to the core of spheroids is limited, and this limitation becomes pronounced with larger spheroid aggregates as it forms a diffusion gradient [23]. Additionally, despite the various techniques utilized in spheroid formation, each has its own unique challenges. For example, the hanging drop method is a simple method to implement and provides uniform spheroid shapes with greater control over spheroid shapes. However, it is tedious to handle and time-consuming, and inefficient due to low throughput. In other spheroid formation techniques, long-term survivability and tedious media exchange are the key challenges [28]. Despite exhibiting a 3D structure, inherently, spheroids lack the complex architecture of tissues in vivo and, therefore, cannot completely recapitulate the physiological environment. 

#### 2.1.2. Organoids

Organoids are 3D self-aggregating assemblies containing multiple cell types arranged spatially, such as cells in a tissue, recapitulating cellular and molecular stages in early organ development [29,30]. They have been used as tissue models to explore mechanisms of organ development. Organoids are increasingly being used in medical research, specifically in preclinical studies and in 3D tissue models, to study cellular interactions and drug-toxicology, pharmacology, and microbiology [29]. The 3D architectural and functional similarities to the tissue of origin make organoids an excellent model for studying complex cell–cell interactions and tissue development. The fabrication of organoid models is similar to the processes involved in the generation of spheroids (Figure 2A(i–vi)) [27,31]. However, the key difference is that in organoid formation, pluripotent stem cells and embryonic stem cells are given specific signaling cues that act as instructions to form 3D organoids of a variety of tissues [31]. Organoids have been employed in the generation of optical cups, liver, brain, lung, and heart [32,33,34,35,36]. They have also been used to model disease conditions to study disease development and progression. For example, in a recent study by Richards et al., cardiac organoids with oxygen-diffusion gradients were fabricated to model the human heart after myocardial infarction while recapitulating the hallmarks of myocardial infarction [36]. Yang et al. developed a mice 3D testicular organoid using testicular cells from BALB/c mice to investigate Zika-virus-induced mammalian testicular damage [37]. The key challenge of using organoids is the lack of vasculature. Optimization of the conditions for incorporating more than one type of cells to mimic in vivo structure is required [38]. Additionally, the effect of ECM composition and cell–matrix interaction requires further investigation to develop robust model systems. While there have been significant advances to overcome this challenge, research into multi-organ communication requires further investigation.

#### 2.1.3. Cell Sheet Engineering

Cell sheet engineering is a form of tissue engineering methodology that does not require a scaffold. In this method, cells are grown in vitro by placing a single-type cell on a stimuli-sensitive polymer (Figure 2B). In a culture environment suitable for cell growth, cells are grown till a three-dimensional cell sheet is generated. By inducing a stimulus such as heat, the polymer becomes hydrophilic, enabling the detachment of the cell sheet from the polymer base [39]. Cell sheet engineering has applications among various organs such as the heart, cornea, bladder, liver, and bone. The key advantage of using cell sheet engineering is the ability to co-culture cells and generate a vasculature network. For example, Sakaguchi et al. observed that endothelial cells within cell sheets spontaneously form blood vessel networks as in vivo capillaries [40]. Wu et al. investigated the therapeutic benefits of cell sheets derived from umbilical cord mesenchymal stem cells on rat models with induced ischemic heart failure [41]. The authors subjected H9C2 cardiomyocytes under hypoxia conditions and starvation to observe cell apoptosis as a 2D model, and an ischemic model was made by subjecting rats with Left Anterior descending artery (LAD) ligation to induce ischemic conditions [41]. The study observed that the cell sheets improved cell retention in the myocardium affected by ischemic heart failure, improved cardiac function, attenuated cardiac fibrosis, and induced neovascularization [41]. While recent research indicates that cell sheet engineering may pose a viable therapeutic solution, a major drawback of this method is the generation of hypoxic conditions within thicker cell sheets. Additionally, the lack of well-developed vascular networks within the cell sheet at the time of the generation of sheets poses further translational limitations [42]. 

### 2.2. Anchorage Dependent (Scaffold Based) 3D Tissue Models

**Figure 3:**
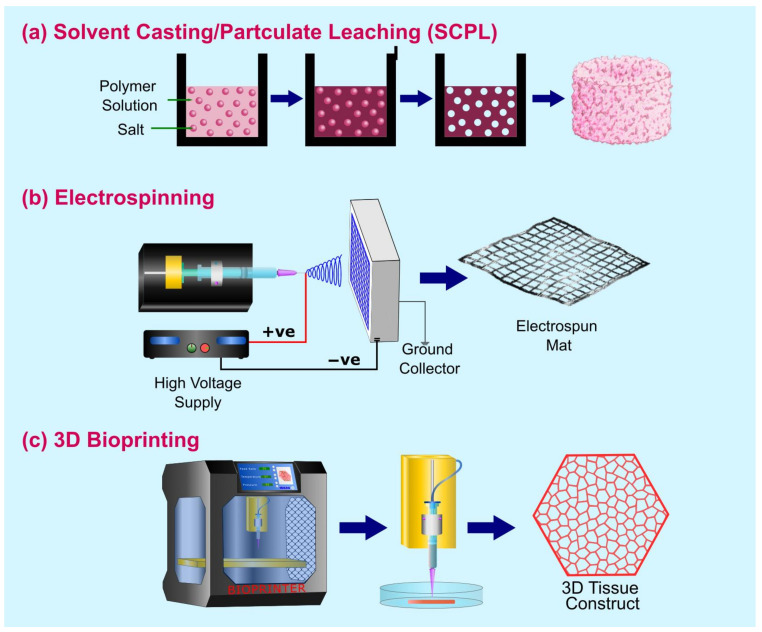
Schematic representation of the methods used in the generation of 3D tissue architecture.

3D tissue models offer the versatility of generating mini-organs that mimic in vivo physiology of a specific tissue. However, these models do not completely recapitulate the characteristics of the tissue. Spheroids and organoids have major drawbacks, such as poor mechanical strength and closed 3D geometry. This results in decreased oxygen and nutrients delivery to the center and hampers the use of conventional assays and instrumentation for screening studies such as nutrient and oxygen transport, absorption kinetics of drugs, and cell–cell interactions [43,44]. The paradigm of tissue engineering involves the conglomeration of living cells within bioartificial support to generate a 3D living structure with mechanical, structural, and functional properties equivalent to human tissue [45]. While the generation of artificial constructs is primarily for regenerative purposes, artificial tissues are being developed to replace reliance on animal models, which are dissimilar to human physiology and do not provide accurate predictions for human tissue responses. The conventional methods, from the perspective of tissue engineering for regenerative purposes, rely on the generation of support structures that act as a temporary scaffold to aid tissue regeneration while gradually degrading and being replaced by autologous tissues [46]. However, from the perspective of modeling, tissue replication should be designed to recapitulate the specific conditions being mimicked. This process is extremely complex due to several factors involved in the mimicking of tissue. Specifically, each tissue exhibits varying features such as porosity, ECM composition, cell phenotypes, and signaling pathways [47]. Ergo, the fundamental elements to consider in the designing of artificial tissue are the material for scaffolds, the cell source, the chemical stimuli, and the method for generating the correct tissue architecture. Additionally, it is pertinent that the choice of material is significantly dependent on the tissue being mimicked as the material will form the ECM, and therefore, the scaffold must meet the specific mechanical, chemical, physical, and biological requirements to achieve cell diffusion, proliferation, viability, and functionality [46]. The key modalities used in the generation of scaffolds for 3D tissue models are Solvent Casting Particulate Leaching (SCPL), Electrospinning, and 3D Bioprinting. Figure 3 provides a schematic representation of the methods, and Table 2 highlights the advantages and disadvantages each method has to offer. 

#### 2.2.1. Solvent Casting Particulate Leaching (SCPL)

SCPL is a popular technique used in the fabrication of highly porous polymer scaffolds for hard tissues such as bone and teeth. In this method, a salt that is insoluble in the polymer is admixed in a polymer solution followed by an evaporation process to remove the solvent, resulting in a salt-polymer composite. The composite matrix is then submerged in water to leach out the salt resulting in a highly porous structure (Figure 3a) [48]. Through this method, 50–90% porosity is achieved [49]. A key advantage of this method is the relative ease and low cost associated with the fabrication of highly porous and tunable pore size that enables the migration of cells within the scaffolds [50]. Similar processes that are employed in the generation of highly porous structures are freeze-drying [51,52], thermal-induced phase separation (TIPS) [53], and gas foaming [54]. The advantages and disadvantages of this method are covered in Table 2. 

#### 2.2.2. Electrospinning

The term is derived from electrostatic spinning and is a method that utilizes a high-voltage electric field to draw charged threads of ultrafine nanometric scale fibers from polymer melts or solutions [55,56]. The technique is complicated and involves a process where a charged droplet of polymer in a liquid phase under high voltage results in an electrostatic repulsion counteracting surface tension and elongation of the droplet to a critical point of liquid stream eruption termed a Taylor cone [56]. As shown in Figure 3b, a standard electrospinning system consists of a syringe pump, a metallic needle, a high-voltage DC supply, and a grounded collector. In the process of electrospinning, solvents evaporate, and the resulting fibers are solidified to form nonwoven fibrous membranes. Typically, cells suspended in cell culture media are seeded on electrospun mats in tissue culture well plates to cultivate cells within the scaffold [57]. Recently, there have been advances in incorporating cells within the polymer solution as a cell-laden bioink to generate cell-laden fibrous structures [39]. This technique was first introduced by Townsend-Nicholson et al., who used a coaxial system to encapsulate cells in a bio-suspension within an outer core of PDMS [40]. The key material and process parameters that need to be considered in the generation of either electrospinning or cell-electrospinning are viscosity, applied electric field, feed rate, and the distance between the nozzle and collector plate, along with environmental factors such as room temperature, relative humidity [39]. Table 2 summarizes the advantages and disadvantages of using such a system. 

#### 2.2.3. Bioprinting

**Figure 4:**
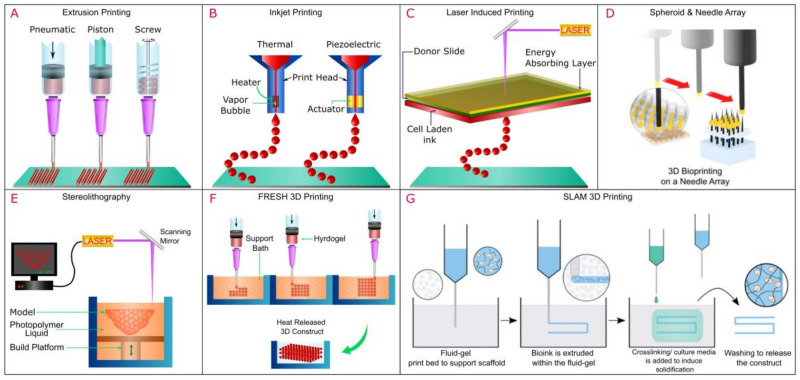
Modalities of 3D Bioprinting (**A**) Extrusion based printing (**B**) Inkjet Printing (**C**) Laser Induced Printing (**D**) Kanzen Spheroid and needle array (**E**) Stereolithography (**F**) FRESH 3D printing method (**G**) SLAM 3D Printing. (**D**) reproduced under the terms and conditions of the Creative Commons CC BY 4.0 License [64] Copyright 2017, The Authors. Published by Springer-Nature Publishing. G reproduced under the terms and conditions of the Creative Commons CC BY 4.0 License [61] Copyright 2019, The Authors. Published by Advanced functional materials.

**Figure 5:**
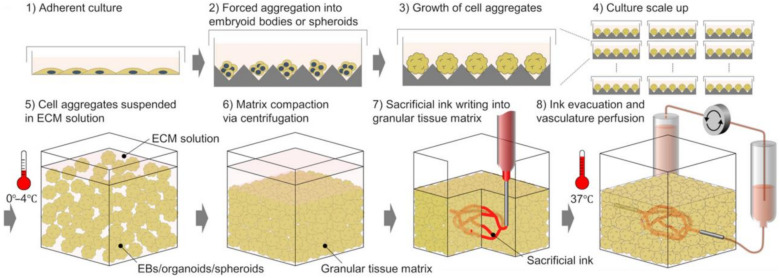
Schematic representation of the SWIFT process. Copyright © 2023 The Authors [62], some rights reserved, exclusive licensee American Association for the Advancement of Science. Distributed under a Creative Commons Attribution Non-Commercial License 4.0.

3D bioprinting is the layer-by-layer deposition of cell-laden biomaterials in 3D space based on a predetermined geometry. Complex geometries and shapes are designed through computer-aided design (CAD) software or geometries extracted from medical images. The main modalities of 3D bioprinting are based on the delivery system of the cell-laden biomaterials termed bio-inks and include extrusion-based (extrusion can be achieved via pneumatic, piston, or screw), inkjet (thermal or piezoelectric), and laser-assisted [58] (Figure 4). In a typical extrusion-based 3D bioprinting system, bioink is extruded via a needle, and based on the pattern generated in a CAD file, a 3D structure in a bottom-up approach is generated (Figure 4A). Three-dimensional bioprinting is a rapidly evolving technology employed to print a variety of tissue structures of various organs, and the frontier of 3D bioprinting is the printing of a complete artificial whole organ, which was most recently achieved by Mirdamadi et al. [59] using a novel technique termed Freeform Reversible Embedding of Suspended Hydrogels (FRESH). In the study, the authors modified an extrusion-based bioprinter and embedded alginate in a support bath comprised of gelatin microparticles suspended in a calcium chloride solution [59]. The core principle is that the gelatin microparticles act as a support bath with multiple crosslinking strategies to gel the different types of hydrogels while providing support for embedded hydrogels that would normally collapse in conventional additive manufacturing processes as they are being printed (Figure 4F) [60]. Senior et al. modified the FRESH bioprinting approach to generate stable hydrogels with low viscosity, termed Suspended Layer Additive Manufacturing (SLAM) [61]. In their study, bioinks with low viscosity in the liquid phase prior to gelation were extruded in an agarose gel that exhibited shear thinning property as the material was extruded and regained its structure upon removal of the shear force entrapping the suspended hydrogel [61]. A crosslinker was then allowed to diffuse through the agarose fluid gel, which resulted in the hydrogel forming stable structures and could be easily removed from the fluid gel [61] (Figure 4G). While microgel support baths have been used to demonstrate full organ printing [59], a shift from this paradigm is the bioprinting of a sacrificial bioink within a slurry-support bath comprised of cellular spheroids in a technique termed sacrificial writing into functional tissue (SWIFT) (Figure 5). Skylar-Scott et al. reported the use of this technique to generate a living matrix primarily composed of tightly compacted tissue-specific organ building blocks from iPSC-derived embryoid bodies, multicellular spheroids, or organoids [62]. Within the living matrix, a sacrificial ink is patterned and embedded via 3D printing, which, when removed, yields perusable branching channels and conduits, thereby resembling vascularized networks (Figure 5) [62,63]. While Support bath systems with extrusion-based bioprinting could be an effective platform in the fabrication of microtissues, however, controlling the position within the 3D space is a challenge [63]. Novel methods to circumvent these challenges are emerging within the scientific community and have been covered elsewhere [63].

3D bioprinting offers versatility in controlling essential parameters such as bioink composition, printing speed, needle gauge, extrusion pressure, and scaffold geometry. However, despite the plethora of biomaterials available for this technique (Table 3), each biomaterial has unique properties that must be optimized to generate suitable constructs. A major benefit of this method is the ability to generate a complex vasculature network via bioinks laden with endothelial cells, as shown in recent research by Noor et al. [65]. Despite the large library of biomaterials that can be used, not all materials have gelling properties required to hold the shape fidelity of the final printed structure and need to be modified to enhance mechanical strength along with chemical, physical, and biological properties. In certain circumstances, bioinks are stabilized through post-processing crosslinking mechanisms via photon activation through UV light in the presence of a photoinitiator or via ionic crosslinking in the presence of divalent cations. While 3D bioprinting offers vast opportunities, it is severely limited by the availability of printers capable of printing whole organs [66]. Additionally, further research into improving the print resolution of the printed construct and encapsulation of cell densities from a clinical translation outlook remains a challenge [67]. 

#### 2.2.4. Organ-on-a-Chip

The process of developing novel drugs and medical interventions requires the use of in vitro modeling, followed by animal studies, to test the safety and efficacy of newly developed drugs before testing on humans. However, animal models do not provide accurate predictions for human responses. Clinical trials are time-consuming and not cost-effective in the long run. Most novel drugs fail in clinical trials, and therefore, there is a need to develop a system or model that mimics human physiology, remains cost-efficient, and has the capability to provide accurate data. In contrast to biological approaches to generate 3D tissue models, organ-on-a-chip (OOC) systems are used to recapitulate tissue and organ structure by leveraging microfluidic physics along with microfabrication engineering techniques and biomaterials to create micro-physiological systems that model tissue structure and disease conditions. Research into the development of microfluidic channels to study signal pathways, drug responses, and tissue functions is ongoing [68]. For example, Zhao et al. employed OOC to create a platform to generate chamber-specific cardiac tissue and disease modeling to measure contractile force in ventricles and atriums and their response in the presence of drugs [69]. Similarly, Parsa et al. developed a platform to study mechanisms of cardiac hypertrophy with low cell volume [70]. 

**Figure 6:**
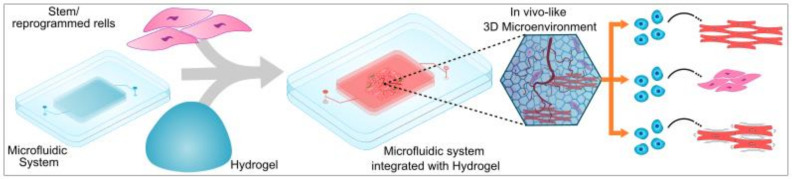
Microfluidic system integrated with hydrogels and cells to provide in-vivo-like 3D microenvironment with biochemical and biophysical cues that result in enhanced differentiation of stem cells or reprogrammed cells, generate functionally mature tissue specific cells and enable a structurally organized microenvironment.

**Figure 7:**
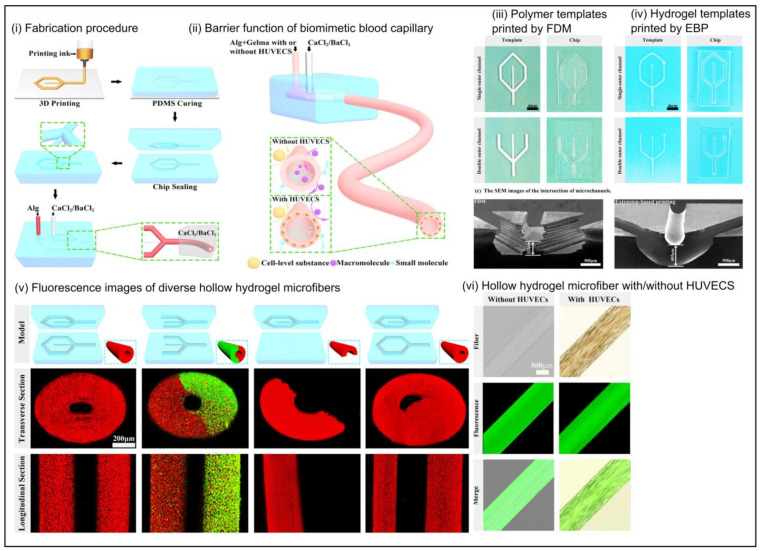
Fabrication of microfluidic chip to generate an in vitro model to simulate hollow biomimetic capillary using 3D bioprinting and hydrogel. (**i**) fabrication of templates and microfluidic device (**ii**) simulating hollow blood capillary. Comparison of templated printed using 3D printing, with (**iii**) fused deposition model (FDM) and (**iv**) using hydrogels and extrusion-based bioprinting. (**v**) fabrication and characterization using fluorescence microscopy of diverse hollow structures (**vi**) barrier function of hollow hydrogel microfiber with cells. Image reprinted with permission from [79] Copyright © 2023 by the authors ACS Biomaterials Science and Engineering.

There is a wide range of organ systems that have been modeled on an OOC platform, including the heart [71], kidney [72], brain [73], lung [74], intestine [75], liver [76], and eyes [77]. Additionally, OOC has been employed to study tissue-specific diseases. Costa et al. reported the use of microfluidics to mimic arterial thrombosis in vitro [78]. The study was designed to replicate a three-dimensional architecture of coronary arteries under healthy and stenotic conditions by modeling healthy and stenotic arteries to create a microfluidic chip with inlets and outlets to allow perfusion through the system [78]. This enabled the authors to study the effect of shear rates within arteries and enable a better understanding of arterial thrombosis [78]. Microfluidic technology has also been leveraged as a tool to generate spheroids and organoids [26], study drug pharmacokinetics, and the generation of micro bioreactors where 3D bio-printed tissue constructs can receive oxygen and nutrients under laminar flow conditions. While most microfluidic systems use a design-based approach and leverage fluid behavior on a microscale, the lack of ECM or an in-vivo-like microenvironment is a drawback in OOC. OOC technology is based on the use of soft lithography to generate molds of microchannels with the use of polydimethylsiloxane (PDMS) as a substrate material. The high resolution offered by stereolithography and the ability to miniaturize the microenvironment enables researchers to study complex diseases and their behavior in a heterogeneous environment. In designing and production of OOC, the selection of cells and biomaterials must be given extensive consideration. In order to improve the relevance of OOC, it is crucial to include vascular networks that can provide efficient nutrient and oxygen diffusion across the tissue or microfluidic channels. There has been a focus on the incorporation of scaffolds or hydrogels into microfluidic systems to overcome this drawback. The presence of an ECM-like matrix to house cells provides both biophysical and chemical cues that aid in the development of a more in-vivo-like microenvironment. Figure 6 provides a schematic representation of the microfluidic system integrated with hydrogels to generate 3D in vitro models to study disease. For example, Shang et al. used 3D bioprinting to generate biomimetic hollow blood capillaries [79]. The authors created microchannels using 3D printing and injected a composite of GelMA and Alginate incorporated with human umbilical cord endothelial cells (HUVECS), the hydrogel being crosslinked with either barium or calcium chloride Figure 7i and studied the proliferation of cells in the hollow chamber [79]. Hong et al. used 3D bioprinting to fabricate cancer spheroids for evaluating the drug resistance of cancer cells [80]. In their study to evaluate the efficacy of drug resistance of cancer cells, the authors printed 3D mini-wells using poly (lactic acid) in a grid structure. The authors then embedded drug-resistant MCF-7 breast cancer cells in a gelatin–alginate hydrogel bioink and 3D bioprinted into the mini-wells to encourage single spheroid formation [80]. The use of hydrogels encapsulated within microfluidic devices to provide a more comprehensive in-vivo-like environment could change the research field towards lesser reliance on animal models. 

Despite the significant advantages this technology has to offer, there are several challenges that need to be addressed. For example, PDMS is the most common material used as a substrate to build a microfluidic device. However, it is known that PDMS absorbs small molecules such as drugs and may have an impact on drug bioactivity in OOC devices designed to study cell behavior and drug efficiency [81]. While there are other materials that can be used in the generation of microfluidic devices, PDMS is one of the most predominantly used materials that is used in the generation of microfluidic devices, and these materials have been thoroughly reviewed elsewhere [82]. The lack of multi-organ interaction and communication is a drawback of this technology. However, researchers have reported the generation of multi-organ/human-on-a-chip. Abaci et al. reported a conceptual study on the design parameters and considerations in developing such a model [83]. The benefits offered by OOC technology outweigh the drawbacks, which have resulted in the continued development of this technology. With the incorporation of novel biomaterials and nanotechnology, OOC platforms are expected to evolve with technological advancements in the future.

## 3. Biomaterials for 3D Tissue Modelling

Advances in research have led to the development of improved 3D tissue models for in vitro studies. Cells in nature reside in a molecular matrix composed of protein, glycosaminoglycan, and glycoconjugate, termed the extracellular matrix (ECM). The ECM provides physical scaffolding, biochemical cues, and mechanical stability to cells and is necessary for morphogenesis and homeostasis [84]. The engineering of ECM that mimics native tissue matrix begins with the identification of a biomaterial that is critical in the formation of a scaffold. The choice of biomaterial is dependent on the tissue being modeled. Biomaterials are based on three categories (a) Polymers, (b) metallic, and (c) ceramics. Factors that influence the choice of materials are the type of tissue being mimicked, structural integrity, adequate mechanical environment, bioactivity, biocompatibility, and biodegradability [84]. The biomaterial should provide structural support for cellular attachment, growth, proliferation, and migration while consisting of adequate mechanical properties and an environment native tissue matrix provide to cells. Materials should be bioactive and biocompatible to provide bioactive cues and growth factors while reducing the risk of immunological response in the presence of an artificial scaffold. Additionally, the scaffold or matrix should act as a support structure facilitating correct localization and retention at the site of tissue damage [85]. While biodegradability is key for the formation of the vascular network and allows for patients’ own ECM to replace the scaffold and degrade over time without any cytotoxic effects [86], this factor is organ-specific. For example, in regenerative medicine for hard tissues such as bone or teeth, materials are engineered from metallic or ceramic biomaterials to reduce the rate of biodegradability. Table 3 provides a summary of the various biomaterials and their pros and cons. 

**Table 3:** List of Biomaterials, both natural and synthetic employed in tissue engineering and their advantages and disadvantages.

Biomaterial	Type	Pros	Cons	Ref
**Collagen**	Natural	High biocompatibility, biodegradable, high cell adhesion, and cell remodeling. Has high printability, is biocompatible, low immunogenicity	Poor mechanical properties, unpredictable degradation in vivo, high thrombogenic potential	[87]
**Gelatin**	Natural	Cheap, biocompatible, easy to modify, good proliferation, biodegradable	Brittleness, low mechanical properties, fast degradation	[88]
**Chitosan**	Natural	Biocompatible, biodegradable, high cell proliferation	Lower mechanical properties, immunogenic	[89]
**Fibrin**	Natural	High cell adhesion and viability, quick gelation and good cell migration, and vascularization	low printability, biocompatibility, low mechanical strength	[90]
**Hyaluronic Acid**	Natural	Biocompatible, biodegradable, high cell proliferation and viability, high printability	low mechanical strength	[91]
**Alginate**	Natural	Biocompatible, biodegradable, sustained release, adoptable mechanical strength with cell growth, rapid gelation	low cell adhesion	[92]
**Pectin**	Natural	Cheap, biocompatible, can be modified, plant derived, good cell proliferation, biodegradable	Poor mechanical properties, Slower gelation time	[93]
**Decellularized** **ECM**	Natural	Keeps vasculature network intact	Variation caused by different decellularization methods,	[94]
**Starch**	Natural	Cheap, biocompatible, versatile rheology,	Poor mechanical properties, slower gelation time, needs high temperature (70–90 °C) to gelatinize, at higher temperatures, phase separation between composite materials may occur	[95]
**Fucoidan**	Natural	Good bioactive properties, biocompatible, biodegradable, used to enhance properties of other natural biomaterials	Does not gel on its own, crosslinking strategies need to be optimized, high synthesis cost	[96]
**Silk Fibroin**	Natural	Biocompatible, good mechanical properties	High cost of production,	[97]
**Hydroxyapatite**	Natural/Synthetic Synthesis	Bioactive, biocompatible, hydrophilic,	brittleness, low tensile strength and fracture toughness	[98,99]
**Polycaprolactone (PCL)**	Synthetic	Moderate mechanical strength. Biocompatible	Slow degradation, lower cell adhesion/aggregation, hydrophobic, inflammation due to acid degradation products	[100]
**Poly Lactic-co-Glycolic Acid (PLGA)**	Synthetic	Biocompatible, biodegradable, immunogenic	Brittle and relatively hard, lower cell adhesion/aggregation, inflammation due to acid degradation products	[101]
**Poly(itaconate-co-citrate-cooctanediol) (PICO)**	Synthetic	Biocompatible, biodegradable, cheap, good mechanical properties, fast crosslinking, non-cytotoxic to cells	UV cross linking	[102]
**Poly (ethylene glycol)** **(PEG)**	Synthetic	Biocompatible, biodegradable, can be modified with various functional groups	Moderate mechanical strength, low printability, difficulty in scalability, Lower cell adhesion	[92]
**Polyphosphazenes**	Synthetic	Biocompatible, good mechanical properties, slow degradation (hard tissues)	Slow degradation (soft tissues)	[97,103]
**Polyurethanes**	Synthetic	Good mechanical properties, good rheological properties	Poor degradability, copolymerization is required	[104]
**Polyanhydrides**	Synthetic	Good flexibility, controllable degradation rates	Weak mechanical properties	[104]
**Poly(propelene-fumarate)**	Synthetic	Good processability, good ductility, biocompatibility, easily forms covalent polymer networks	Challenging to handle the material due to high viscosity, increased cytotoxicity and acute inflammation, variation in molecular weight between crosslinking agents	[105,106]
**Metals**	Synthetic	Biocompatible with good mechanical properties, low degradability (Tissue dependent)	Subject to oxidation, low degradability (Tissue dependent), may be cytotoxic due to release of free metal ions	[107]
**Ceramics**	Synthetic	Osteoinductive and osteoconductive in bioactive ceramics, low toxicity, biocompatible, angiogenetic potential,	High brittleness, weak, low bioactivity	[107]

### Characterization and Optimization of Biomaterials

There is a plethora of biomaterials available in the generation of ECM, such as structures, and the choice of biomaterial is highly dependent on the tissue of interest. On the formation of stable tissue-like constructs through any of the biofabrication techniques, the constructs should be subjected to various characterization techniques to ensure that they meet the parameters as close as possible to native tissue. Table 4 highlights the fundamental properties and the quantitative methods utilized in the characterization of these properties of biomaterials. 

## 4. Cell Sources

**Figure 8:**
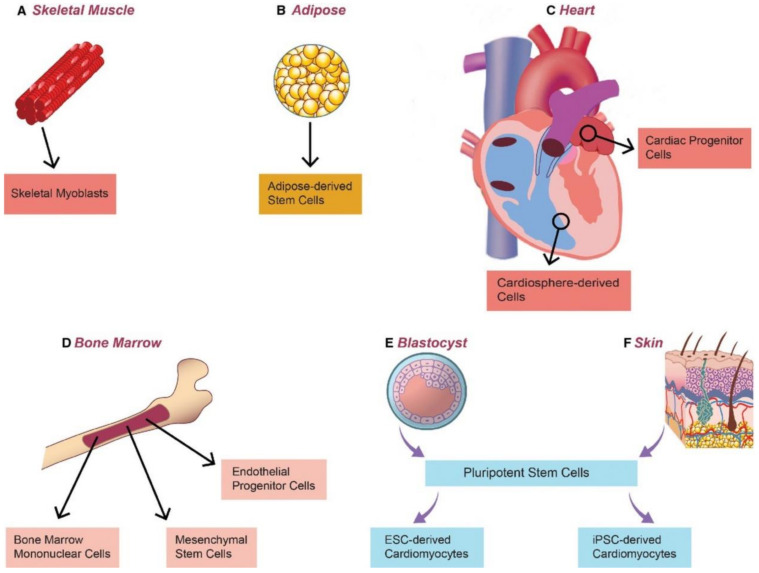
Schematic representation of the Cell Sources. (**A**) skeletal myoblasts, (**B**) adipose-derived stem cells, (**C**) cardiac ‘progenitor’ cells and cardio sphere-derived cells (**D**) bone marrow-derived stem cells (**E**) Embryonic stem cells derived from the blastocyst (**F**) induced pluripotent stem cells derived from skin. Modified and adapted with permission from [113] under creative commons license CC BY 4.0. Copyright 2015 The Authors. Journal of Cellular and Molecular Medicine published by John Wiley & Sons Ltd. and Foundation for Cellular and Molecular Medicine.

The incorporation of cells is essential in the generation of functional 3D tissue models. In general, cells can either be seeded on an existing carrier matrix or can be encapsulated within a biomaterial [108]. The factor governing cell incorporation is dependent on the tissue architecture fabrication method. While there are various methods to create scaffolds for 3D tissue models, the choice of the cell is highly dependent on the tissue being modeled. Primary cells closely mimic in vivo physiological state of the tissue or organ of interest; however, not all organs or tissues have primary cells in sufficient quantities or have limited proliferative potential. Figure 8 provides a schematic representation of the various cell sources [108,109,110,111,112].

Recent findings on the differentiation of stem cells towards any tissue-specific lineages have led to significant advancements in tissue modeling and have been reviewed exhaustively elsewhere [108,111,112]. The conglomeration of novel biomaterials fabrication strategies, advances in stem cell biology, and 3D bioprinting has evolved as a next-generation technology for in vitro tissue model development. Table 5 provides a summary of the advantages and disadvantages of the various cells used in 3D bioprinting.

## 5. Imaging Modalities of 3D Tissue Models

**Figure 9:**
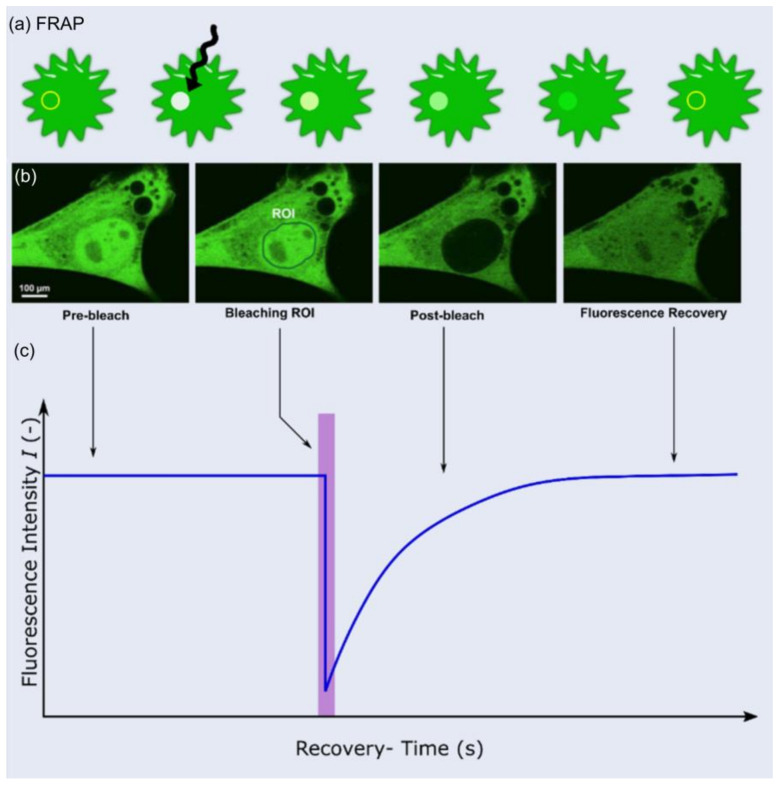
(**a**) Schematic representation of FRAP method, (**b**) Example of a FRAP experiment (**c**) Anatomy of a typical FRAP curve. Modified and reprinted with permission from [123] under creative commons CC BY 4.0. Copyright © 2023 by the authors; licensee MDPI, Basel, Switzerland.

There are a variety of methods used in the generation of 3D tissue models which have been discussed. While characterization methods are employed to ensure that the physical, mechanical, chemical, and biological parameters are met, these methods are often destructive and do not provide an insight into what is happening in the tissue once it is constructed. Therefore, additional methods are required to characterize and ensure that the final tissue model works as intended. In 2D cell culture and model systems, imaging, molecular, and immunohistochemistry techniques are commonplace. However, in a 3D system, advanced techniques are essential. Imaging techniques allow observation of the live-cell morphology and other organelles within the cells from 3D tissue models. Scanning electron microscopy analysis helps to find the cell morphology, migration, attachment, and cell–cell and cell–matrix interaction. Recent techniques have shown the real-time analysis of biological parameters in 3D cell/tissue models. Ruslan et al. used polymer-conjugated nanoparticles to identify O_2_ in cells present in the 3D tissue models [116]. Muller et al. used nanoparticle-based fluroionophore to study live analysis of K^+^ flux in 3D tissue models and animals [117]. Cell density can be analyzed with the presence of nucleated cells with H&E-stained histologic section photographs by using the ImageJ tool [118]. Table 6 provides a summary of the common advanced imaging techniques used for the analysis and examination of 3D tissue models, and Figure 9 provides the use of Optical Coherence Tomography (OCT) to characterize hydrogels.

**Table 6:** Common advanced imaging techniques used to analyze 3D tissue models and live cells within scaffolds.

Imaging Modalities	Characteristics	Application	Ref
**Fluorescence**	Cells are marked with fluorescence markers and a sample is irradiated with wavelengths between visible and ultraviolet to reveal fluorescent species.	Cell viability, proliferation	[119]
**Confocal Imaging**	Advanced version of fluorescence resulting in high-resolution images by collecting light from a single plane of focus and eliminating out-of-focus light	Cellular Structure, viability, live imaging, 3D reconstruction	[120]
**Scanning Electron Microscope (SEM)**	A technique used to produce high-resolution images of surface topography by scanning with electrons on the surface	Surface morphology	[121]
**Transmission Electron Microscope (TEM)**	A technique in which electrons pass through ultrathin samples to generate high-resolution images	Characterization of pore structure, nano structures	[122]
**Fluorescence Recovery After Photobleaching (FRAP)**	A high-intensity laser causes bleaching in a region of interest (ROI) and gradual recovery of fluorescence from the surrounding environment to the bleached area is observed.	Used to study oxygen and nutrient diffusion across cells and tissue structures.	[119]
**Fluorescence Loss in Photobleaching (FLIP)**	Involves repeated bleaching of an ROI and measuring fluorescence intensity outside the bleached area where a drop in fluorescence intensity due to bleached non-fluorescent molecules provides quantitative data on molecular mobility.	Molecular mobility, exchange of molecules between cell compartments	[123]
**Fluorescence localization after photobleaching (FLAP)**	Involves labeling molecules with two fluorescent labels: one to be bleached locally and the second is a reference label that remains intact. By measuring the difference between bleached and unbleached signals gives an absolute FLAP signal which can be used to track the labeled molecule.	Ability to identify molecules and populations that have varying speeds and have dissimilar dynamics.	[123]
**Fluorescence Resonance Energy Transfer (FRET)**	A physical process in which a molecular fluorophore is excited and a nonradiative energy transfer occurs to another fluorophore through intermolecular long-range dipole-dipole coupling. This process is highly dependent on the distance between two fluorophores.	Live -cell analysis of cell biology, cellular interaction in 3D scaffolds, Protein–protein interaction, receptor activation, intramolecular distances	[124,125]
**Fluorescence Lifetime Imaging Microscopy (FLIM)**	A method in which the fluorescence decay time is measured. In combination with FRET, this method can be used to map spatial distribution to indirectly measure bimolecular interactions, concentration, and conformational changes.	Measuring intramolecular distances, evaluate therapeutic efficacy in drug screening	[119]
**Phosphorescence Lifetime Imaging Microscopy (PLIM)**	Similar to FLIM; however, this process images phosphorescence quenching.	Measuring partial oxygen concentration and identify hypoxic environment.	[119]
**Optical Coherence Tomography (OCT)**	Measures optical backscatter from different microstructural features within materials and tissues to generate high-resolution images of the cross-sections of tissue.	Quantify changes in porosity of scaffold, pore size, pore interconnectivity, cell dynamics and tissue development.	[126,127]
**Micro-Computerized Tomography (MCT)**	Involves exploiting variations in X-ray absorption, refraction, and scattering to form contrast alterations resulting in spatial distribution of material densities and providing 3D images of the internal structure.	Largely used in bone tissue models, as this technique offers the ability to form contrasts between soft and hard tissues.	[119]

### 5.1. Fluorescence Recovery after Photobleaching (FRAP) Using Confocal Microscopy

Confocal fluorescence microscopy is an advanced method of fluorescence microscopy where high-resolution images can be obtained by the introduction of a spatial pinhole before the light source and the detector [128]. The aperture of the pinhole can be controlled to limit diffraction and thereby eliminate out-of-focus light from the sample. FRAP is a method used to study the movement of molecules that have been doped with a fluorescent dye (Figure 9) [123]. In FRAP, mobile fluorescent molecules are bleached by a high-intensity laser source. The bleached molecules are exchanged with fluorescent molecules from the surrounding area resulting in a recovery of fluorescent intensity. This information is plotted on a recovery curve and can be used to study the behavior of the molecules (Figure 9) [129]. The key advantage of using FRAP with confocal microscopy is that a small region in high resolution can be observed. For example, it is possible to study oxygen diffusion in a scaffold. Lee et al. used this method to examine the microscale diffusion of oxygen in scaffolds generated via electrospinning [130]. By introducing simulated cell concentrations, the study reports the ability to predict the efficiency of the scaffold. However, this technique requires further standardization protocols to be established as a viable method to characterize 3D tissue models. 

### 5.2. Optical Coherence Tomography (OCT)

**Figure 10:**
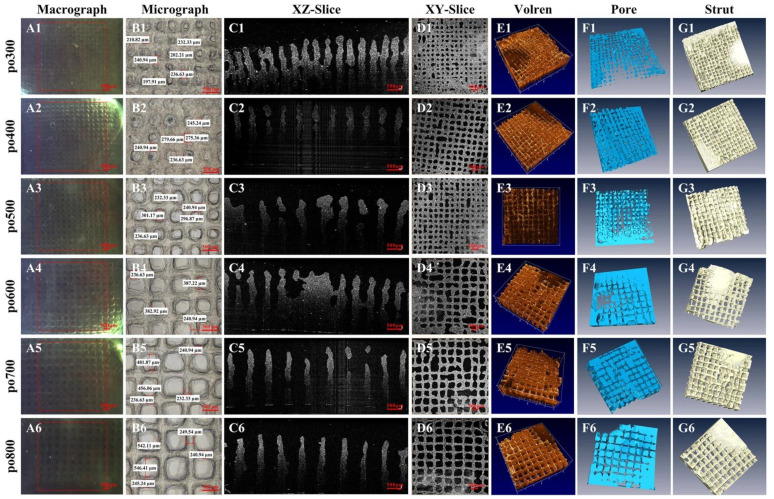
Cell-laden 3D bioprinted structures with varying pore size characterized using OCT (**A1**–**A6**) Macrographs (**B1**–**B6**) Micrographs, (**C1**–**C6**) C OCT Cross-sectional images to a depth of 3 mm. (**D1**–**D6**) en-face OCT images. (**E1**–**E6**) rendering in 3D and (**F1**–**F6**,**G1**–**G6**) 3D reconstruction of hydrogel exhibiting variation in pore size. Image reprinted with permission from [132] under the creative commons license CC BY 4.0. Copyright © 2023 by the authors; Scientific Reports [132].

OCT is a type of imaging modality that performs high-resolution, cross-sectional imaging of microstructures in biological materials by measuring optical backscatter from different microstructural features within materials and tissues [126]. OCT can be used to observe the spatial and temporal changes of these features in real-time and in three dimensions, allowing the screening, identification, and optimization of parameters that govern the usability of tissue [131]. A key feature of OCT is capturing details in high resolution between 15–20 µm depths, thereby allowing the ability to observe scaffold architecture in intricate details [131]. The characteristics of scaffold architecture include parameters such as porosity, pore size, and degree of pore interconnectivity, which influence cellular activity, including cell adhesion, distribution, and proliferation [119,131]. A non-destructive method, OCT imaging, can be used to quantify changes in porosity as the scaffold degrades and cellular growth profile. For example, Zheng et al. used OCT to demonstrate the importance of OCT in the reconstruction of scaffold architecture and cell adhesion by capturing high-resolution images of two scaffolds with different seeding densities of human embryonic kidney cells [131]. Their study concluded that OCT is a viable method that can be used to optimize the parameters of scaffolds. More recently, Wang et al. used OCT to capture high-resolution images of the inner microstructures of cell-laden 3D-printed scaffolds. The study incorporated C3A cells in the gelatin–alginate hydrogel with varying pore sizes and utilized OCT to quantify morphological features, including pore size, pore shape factor, volume porosity, and the interconnectivity of the pores, as shown in Figure 10 [132]. Ultimately, this imaging modality has the capability to improve the understanding of the intricate structures, thereby leading to improved scaffold architecture designs, efficiently mimicking in vivo architecture and improving the efficacy of 3D tissue models.

## 6. Other Imaging Modalities

**Figure 11:**
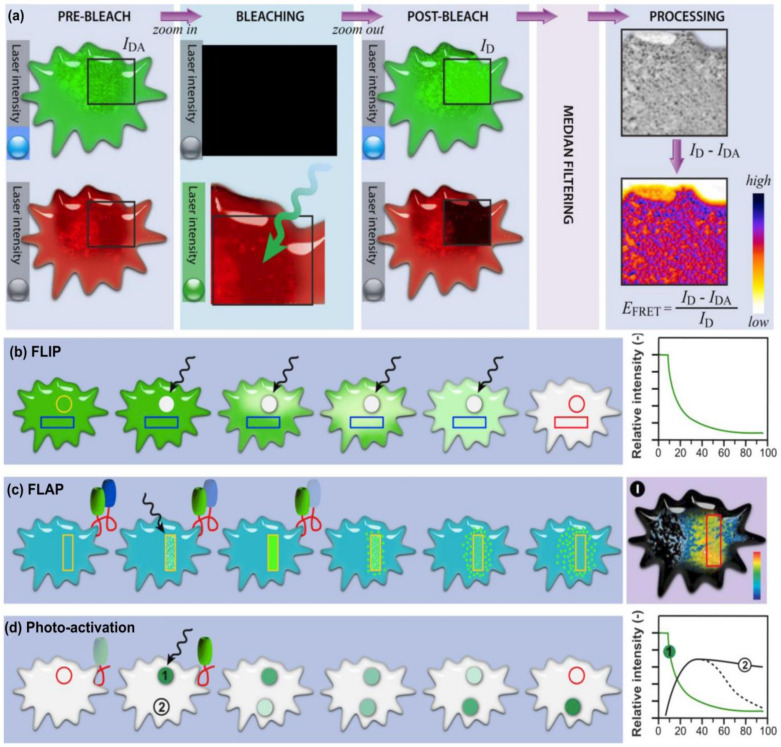
(**a**) workflow of FRET (**b**) Workflow of FLIP, (**c**) workflow of FLAP, (**d**) workflow of Photoactivation. Images reprinted with permission from [123] under creative commons CC BY 4.0. Copyright © 2023 by the authors; licensee MDPI, Basel, Switzerland.

While FRAP and OCT are imaging modalities that can be utilized to perform characterization on 3D tissue models, confocal microscopy imaging provides other methods to characterize and analyze 3D tissue models. Such modalities include Fluorescence Loss in Photobleaching (FLIP), Fluorescence localization after photobleaching (FLAP), Fluorescence Resonance Energy Transfer (FRET), Fluorescence Lifetime Imaging Microscopy (FLIM), Phosphorescence Lifetime Imaging Microscopy (PLIM), and Micro-Computerized Tomography (MCT), and a summary of their characteristics and application can be found in Table 6. Figure 11 provides a workflow of the modalities. Ishikawa-Ankerhold et al. have provided an exhaustive review of the same [123].

## 7. Conclusions and Future Perspective

This review highlights the vast potential of 3D in vitro models for the generation of tissue mimics, disease modeling, and assessment of innovative drugs toward personalized medicine over 2D models. While 2D modeling is a traditional and established method, it lacks the capability to replicate human physiology and diseased conditions. In the context of tissue engineering, the various methods used in the generation of artificial constructs, along with their advantages and disadvantages, are discussed. The potential role of these methods in regenerative medicine is also highlighted. Biomaterials play an important role in the generation of such constructs and models. The choice of biomaterials that have the capability to closely replicate human physiology and promote cellular functions within artificial constructs is critical when considering modeling. With the advent of various stem-cell types, specifically iPSCs (induced pluripotent stem cells), research in disease modeling and personalized medicine has taken an innovative direction. The key advantage of employing the strategy of using 3D in vitro model systems is a reduced dependence on animal models, which are dissimilar to human physiology.

Most reviews discuss the state-of-the-art in tissue engineering research and regenerative medicine; however, methods used in the assessment of artificially generated constructs are a key area that is often neglected. An important aspect of 3D models and tissue engineering is to ensure that the artificial construct has the capability to replicate physiological conditions as closely as possible. Methods such as FTIR, mechanical testing, and biological activity assays to determine cell proliferation and survivability, to name a few, enable researchers to establish artificial tissues as efficient models and maintains standardization from a regulatory perspective. This review provides an exhaustive analysis of the various characterization methods used to evaluate artificially constructed 3D models along with various imaging modalities. Imaging has the capability to provide researchers with a tool to observe the functioning of cells at a microscopic level. It provides a platform where researchers can develop a deeper understanding of the attributes involved in the development and progression of the disease through direct observation. Methods such as optical coherence tomography are used in observing the structure of scaffolds in 3D, while FRAP and FRET can be employed to observe cellular functions.

**Figure 12:**
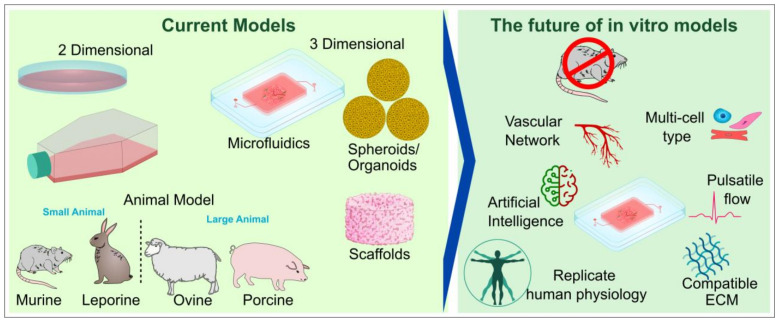
The future of in vitro models: a perspective.

A key challenge with 3D in vitro modeling is that while it has the capability to closely mimic human physiological conditions, it is an incomplete model, hence the reliance on animal models. Towards the future (Figure 12), it is imperative to focus on research towards the development of models that completely considers and mimics various factors and functions within the human body. The choice of biomaterials to have the right cellular microenvironment, appropriate mechanical properties as that of the relevant tissues of interest, the right orientation of cell/s, vascular networks, immune cells, the spatio-temporal release of necessary factors needed for the differentiation or growth of cells, and other factors unique to the tissues of interests, such as conduction properties in case of cardiac and neural tissues. This will allow researchers to work with improved 3D models, develop an improved understanding of diseases, and provide targeted solutions which are easy to manufacture, economically viable, and safe to administer. Furthermore, with the recent implementation of FDA Modernization Act 2.0, we envision that more emphasis will be given to complex and more sophisticated human physiology-relevant 3D in vitro tissue models for drug testing applications in the near future.

## Figures and Tables

**Table 1:** Key differences between 2D and 3D cell cultures for modeling in vivo conditions.

S.No	Characteristics	2D Cell Cultures	3D Cell Cultures	Ref
1	Cell morphology	Cell shape is elongated and grows on a flat 2D surface.	Natural cell shape is preserved with spheroids or organoids structures and with other 3D models	[11]
2	Cell proliferation	Cell growth in 2 dimensions is rapid and does not mimic in vivo	Cell growth is realistic under 3D culture conditions	[12]
3	Cell and ECM interactions	Growing on a flat surface is not mimicking the native tissue environment. There is no cell and ECM interactions.	Cells and ECM interact with each other and make a 3D environment such as the existing interactions in native tissues. These models reduce the cost of in vivo testing.	[1,13]
4	Cell–Cell Interactions	Multi-cell interactions cannot mimic the native organ environment.	Multi-cell interactions in different 3D tissue models can mimic the native environment.	[14]
5	Cell differentiation	Less resemblance to the native tissue.	Mimics native tissue-like differentiation and markers expression are close to the native tissues.	[15]
6	Vasculature	2D co-culture vasculature studies do not mimic the native vascular system.	Ability to incorporate complex vasculature in the 3D model	[16]
7	Protein and gene expression	Lack of 3D culture conditions, the expression levels may not show much resemblance to in vivo	Show resemblance with in vivo environment	[3]
8	Drug response efficacy	Low or sometimes not predictable due to cells growing on plastic substrate	Predictable as structure is—in vivo environment.	[3,17]
9	Apoptosis and viability (tumor models)	Sensitive to study the target drugs	High resistance to the anti-cancer drugs, which replicates the in vivo environment.	[12,18]
10	Mechanical stimulation	Mechanical stimulation may not mimic the native tissue	We can apply mechanical stimulus according to the native environment and it is an accurate representation of cells in vivo.	[14]
11	Physiological relevance	Highly non-relevant to the physiological environment.	Feasible to make physiologically relevant nutritional and oxygen conditions.	[1]
12	Exposure to culture condition	All cells receive nutrients and growth supplements equally.	The core site of the models will not obtain enough nutrients. Different approaches are explored to improve upon the nutrient and oxygen diffusion to the core site.	[13]
13	Experimentation and analysis	Easy to handle and highly reproducible.	Handling is difficult when compared to 2D cultures, less reproducible, and difficult to handle.	[19]
14	Characterizations	Easy to characterize the cells for experiment with any instrument. Well-established characterization techniques are available.	Difficult to characterize the 3D models. Specific instrumentation is required. Time consuming and not so well-established.	[20]
15	Cost	Inexpensive and well established	Expensive and requires further standardization.	[18]

**Table 2:** The advantages and disadvantages of fabrication methods used in creating 3D architectures.

Method	SCPL	Electrospinning	3D Bioprinting
Advantages	Control over pore size and density, ease of fabrication	Highly efficient and well-understood process, in-expensive, controllable fiber dimensions, high resolution	Moderate resolution, diverse choice of biomaterials, dimensional control, cheap, variation in geometry, spatial control of cells deposition.
Disadvantages	Residual solvents and salts, generally isotropic properties, weaker mechanical integrity	3D architecture is challenging, toxic solvents, lower mechanical properties, inhomogeneous cell distribution, time consuming.	Finding the right bio-inks with the right sterilizable and cross-linkable properties. Limited devices can print whole organ, application dependent

**Table 4:** List of quantitative methods utilized in the characterization of the fundamental properties of biomaterials.

Characterization		Properties	Method
**Physical**	Swelling Ratio	Fraction increase in weight of hydrogel due to water absorption	Weighing difference
	Degradation rate	Fractional decrease in material to facilitate tissue growth	Collagenase and Weight measurement
	Porosity and Morphology	Determination of porous structure to facilitate cellular impregnation and proliferation	Scanning Electron Microscope (SEM), Brunauer, Emmett and Teller (BET) technique
**Chemical**	FTIR Spectroscopy	Investigate formation of Chemical Bonds	Standard FTIR Protocol
	H-NMR	Investigate the Molecular Structure	Standard NMR Protocol
	Degree of Functionalization	Quantify functional groups	Habeeb Method
**Mechanical**	Mechanical Loading	Determine elasticity of biomaterials	Youngs Modulus, Tension, Compression, Shear, Torsion, Yield Strength, Ultimate Yield Strength
	Rheology	Determine viscoelastic characteristics such as Shear Thinning, Viscosity, Storage and Loss Modulus	Rheometer, Viscometer
	Printability	Determine optimum parameters to enable efficient printing e.g., Needle Gauge, Print speed, Extrusion Pressure, Geometry, laser power, UV Crosslinking	Based on the tissue architecture equipment
**Biological**	Cell Volume	Optimize quantity of cells required for functional models	Cell Culture methods
	Cell Viability and Proliferation	Determine and monitor the response and health of cells, survivability, and spread tissue model	CCK-8, MTT, XTT, Live/Dead
	Cytotoxicity, Adhesion	Determine toxicity of biomaterials on cells and how well cells adhere to surface of tissue models.	Fluorescence microscopy, Confocal Microscopy
	Immunostaining	Identification and assessment of the topographical distribution of cells, proteins, and detect antigen levels. Eg F-Actin/DAPI	Immunohistochemistry, Flow cytometry, Western Blotting, ELISA, Immuno-electron Microscopy

**Table 5:** Advantages and Disadvantages of the various types of stem cells found within the human body.

Cell category	Pros	Cons	Reference
**Mesenchymal Stem Cells**	Can differentiate into many types of cells	Limited in quantity, differentiation capacity diminishes with age	[113,114]
**Adult stem cell**	Can differentiate into cells of the lineage it belongs to, main function is to repair the organ they are found in	Limited in quantity, expensive Limited capacity to divide	[85]
**Adipose Derived Stem Cells**	Multipotent, easily isolated, easily available	Low survival	[115]
**Embryonic stem cells**	Can differentiate into any cells in the right conditions,	Ethical concerns, allogenic and hence require immunosuppressants	[114]
**Induced Pluripotent Stem Cells**	Can be reprogrammed to embryonic stem cell-like state, can differentiate into any cell,	teratomas formation	[114]

## Data Availability

All the data and materials that support the results or analyses presented in the paper will be made available upon request.

## References

[1] Duval K., Grover H., Han L., Mou Y., Pegoraro A., Fredberg J., Chen Z. (2017). Modeling physiological events in 2D vs. 3D cell culture. Physiology.

[2] Edmondson R., Broglie J., Adcock A., Yang L. (2014). Three-dimensional cell culture systems and their applications in drug discovery and cell-based biosensors. Assay Drug Dev. Technol..

[3] Langhans S. (2018). Three-dimensional in vitro cell culture models in drug discovery and drug repositioning. Front. Pharmacol..

[4] Kusindarta D., Wihadmadyatami H. (2018). The Role of Extracellular Matrix in Tissue Regeneration. Tissue Regeneration.

[5] Gibot L. (2017). 3D tissue models to bridge the gap between cell culture and tissue in assessing electroporation. Handbook of Electroporation.

[6] Ruan J., Tulloch N., Razumova M.V., Saiget M., Muskheli V., Pabon L., Reinecke H., Regnier M., Murry C. (2016). Mechanical Stress Conditioning and Electrical Stimulation Promote Contractility and Force Maturation of Induced Pluripotent Stem Cell-Derived Human Cardiac Tissue. Circulation.

[7] Di Silvio L. (2007). Bone tissue engineering and biomineralization. Tissue Engineering Using Ceramics and Polymers.

[8] Torras N., García-Díaz M., Fernández-Majada V., Martínez E. (2018). Mimicking epithelial tissues in three-dimensional cell culture models. Front. Bioeng. Biotechnol..

[9] Oberman R., Bhardwaj A. (2019). Physiology, Cardiac.

[10] Omar A., Vallabhajosyula S., Sengupta P. (2015). Left Ventricular Twist and Torsion. Circ. Cardiovasc. Imaging.

[11] Baker B., Chen C. (2012). Deconstructing the third dimension-how 3D culture microenvironments alter cellular cues. J. Cell Sci..

[12] Ravi M., Paramesh V., Kaviya S., Anuradha E., Solomon F.P. (2015). 3D cell culture systems: Advantages and applications. J. Cell. Physiol..

[13] Dhaliwal A. (2012). Three Dimensional Cell Culture: A Review. Mater. Methods.

[14] Jensen C., Teng Y. (2020). Is It Time to Start Transitioning From 2D to 3D Cell Culture?. Front. Mol. Biosci..

[15] Griffanti G., Rezabeigi E., Li J., Murshed M., Nazhat S. (2020). Rapid Biofabrication of Printable Dense Collagen Bioinks of Tunable Properties. Adv. Funct. Mater..

[16] Mastrullo V., Cathery W., Velliou E., Madeddu P., Campagnolo P. (2020). Angiogenesis in Tissue Engineering: As Nature Intended?. Front. Bioeng. Biotechnol..

[17] Fang Y., Eglen R. (2017). Three-Dimensional Cell Cultures in Drug Discovery and Development. Adv. Sci. Drug Discov..

[18] Costa E., Moreira A., de Melo-Diogo D., Gaspar V., Carvalho M., Correia I. (2016). 3D tumor spheroids: An overview on the tools and techniques used for their analysis. Biotechnol. Adv..

[19] Kapałczyńska M., Kolenda T., Przybyła W., Zajączkowska M., Teresiak A., Filas V., Ibbs M., Bliźniak R., Łuczewski Ł., Lamperska K. (2018). 2D and 3D cell cultures—A comparison of different types of cancer cell cultures. Arch. Med. Sci..

[20] De Hoogt R., Estrada M., Vidic S., Davies E., Osswald A., Barbier M., Santo V., Gjerde K., Van Zoggel H., Blom S. (2017). Data descriptor: Protocols and characterization data for 2d, 3d, and slice-based tumor models from the predect project. Sci. Data.

[21] Sutherland R., McCredie J., Inch W. (1971). Growth of multicell spheroids in tissue culture as a model of nodular carcinomas. J. Natl. Cancer Inst..

[22] Fennema E., Rivron N., Rouwkema J., van Blitterswijk C., De Boer J. (2013). Spheroid culture as a tool for creating 3D complex tissues. Trends Biotechnol..

[23] Ryu N., Lee S., Park H. (2019). Spheroid Culture System Methods and Applications for Mesenchymal Stem Cells. Cells.

[24] Lim W., Park S. (2018). A microfluidic spheroid culture device with a concentration gradient generator for high-throughput screening of drug efficacy. Molecules.

[25] Utama R., Atapattu L., O’Mahony A., Fife C., Baek J., Allard T., O’Mahony K., Ribeiro J., Gaus K., Kavallaris M. (2020). A 3D Bioprinter Specifically Designed for the High-Throughput Production of Matrix-Embedded Multicellular Spheroids. IScience.

[26] Moshksayan K., Kashaninejad N., Warkiani M., Lock J., Moghadas H., Firoozabadi B., Saidi M., Nguyen N. (2018). Spheroids-on-a-chip: Recent advances and design considerations in microfluidic platforms for spheroid formation and culture. Sens. Actuators B Chem..

[27] Hoarau-Véchot J., Rafii A., Touboul C., Pasquier J. (2018). Halfway between 2D and animal models: Are 3D cultures the ideal tool to study cancer-microenvironment interactions?. Int. J. Mol. Sci..

[28] Benien P., Swami A. (2014). 3D tumor models: History, advances and future perspectives. Futur. Oncol..

[29] Davies J. (2018). Organoids and mini-organs: Introduction, history, and potential. Organoids and Mini-Organs.

[30] Augustyniak, Bertero A., Coccini T., Baderna D., Buzanska L., Caloni F. (2019). Organoids are promising tools for species-specific in vitro toxicological studies. J. Appl. Toxicol..

[31] Velasco V., Shariati S., Esfandyarpour R. (2020). Microtechnology-based methods for organoid models. Microsyst Nanoeng..

[32] Eiraku M., Takata N., Ishibashi H., Kawada M., Sakakura E., Okuda S., Sekiguchi K., Adachi T., Sasai Y. (2011). Self-organizing optic-cup morphogenesis in three-dimensional culture. Nature.

[33] Takebe T., Sekine K., Enomura M., Koike H., Kimura M., Ogaeri T., Zhang R., Ueno Y., Zheng Y., Koike N. (2013). Vascularized and functional human liver from an iPSC-derived organ bud transplant. Nature.

[34] Lancaster M., Knoblich J. (2014). Generation of cerebral organoids from human pluripotent stem cells. Nat. Protoc..

[35] Miller A., Dye B., Ferrer-Torres D., Hill D., Overeem A., Shea L., Spence J. (2019). Generation of lung organoids from human pluripotent stem cells in vitro. Nat. Protoc..

[36] Richards D., Li Y., Kerr C., Yao J., Beeson G., Coyle R., Chen X., Jia J., Damon B., Wilson R. (2020). Human cardiac organoids for the modelling of myocardial infarction and drug cardiotoxicity. Nat. Biomed. Eng..

[37] Yang W., Zhang C., Wu Y.-H., Liu L.-B., Zhen Z.-D., Fan D.-Y., Song Z.-R., Chang J.-T., Wang P.-G., An J. (2022). Mice 3D testicular organoid system as a novel tool to study Zika virus pathogenesis. Virol. Sin..

[38] Joseph J.S., Malindisa S.T., Ntwasa M. (2019). Two-Dimensional (2D) and Three-Dimensional (3D) Cell Culturing in Drug Discovery. Cell Culture.

[39] Hong J., Yeo M., Yang G., Kim G. (2019). Cell-electrospinning and its application for tissue engineering. Int. J. Mol. Sci..

[40] Townsend-Nicholson A., Jayasinghe S. (2006). Cell electrospinning: A unique biotechnique for encapsulating living organisms for generating active biological microthreads/scaffolds. Biomacromolecules.

[41] Wu Y., Zhang H., Wang S., Li L., Wang R., Jiang S. (2022). Human umbilical cord-derived stem cell sheets improve left ventricular function in rat models of ischemic heart failure. Eur. J. Pharmacol..

[42] Owaki T., Shimizu T., Yamato M., Okano T. (2014). Cell sheet engineering for regenerative medicine: Current challenges and strategies. Biotechnol. J..

[43] Lin R., Chang H. (2008). Recent advances in three-dimensional multicellular spheroid culture for biomedical research. Biotechnol. J..

[44] Wilson S., Tocchi A., Holly M., Parks W., Smith J. (2015). A small intestinal organoid model of non-invasive enteric pathogen-epithelial cell interactions. Mucosal Immunol..

[45] Kim M., Evans D. (2005). Tissue Engineering: The Future of Stem Cells. Top. Tissue Eng..

[46] Eltom A., Zhong G., Muhammad A. (2019). Scaffold Techniques and Designs in Tissue Engineering Functions and Purposes: A Review. Adv. Mater. Sci. Eng..

[47] Caddeo S., Boffito M., Sartori S. (2017). Tissue engineering approaches in the design of healthy and pathological in vitro tissue models. Front. Bioeng. Biotechnol..

[48] Sola A., Bertacchini J., D’Avella D., Anselmi L., Maraldi T., Marmiroli S., Messori M. (2019). Development of solvent-casting particulate leaching (SCPL) polymer scaffolds as improved three-dimensional supports to mimic the bone marrow niche. Mater. Sci. Eng. C.

[49] Li Z., Xie M.B., Li Y., Ma Y., Li J., Dai F. (2016). Recent progress in tissue engineering and regenerative medicine. J. Biomater. Tissue Eng..

[50] Sanz-Herrera J., García-Aznar J., Doblaré M. (2009). On scaffold designing for bone regeneration: A computational multiscale approach. Acta Biomater..

[51] Brougham C., Levingstone T., Shen N., Cooney G., Jockenhoevel S., Flanagan T., O’Brien F. (2017). Freeze-Drying as a Novel Biofabrication Method for Achieving a Controlled Microarchitecture within Large, Complex Natural Biomaterial Scaffolds. Adv. Healthc. Mater..

[52] Anandan D., Stella S.M., Nambiraj N.A., Vijayalakshmi U., Jaiswal A. (2018). Development of mechanically compliant 3D composite scaffolds for bone tissue engineering applications. J. Biomed. Mater. Res. Part A.

[53] Martínez-Pérez C.A., Olivas-Armendariz I., Castro-Carmona J.S., García-Casillas P.E. (2011). Scaffolds for Tissue Engineering Via Thermally Induced Phase Separation. Advances in Regenerative Medicine.

[54] Dehghani F., Annabi N. (2011). Engineering porous scaffolds using gas-based techniques. Curr. Opin. Biotechnol..

[55] Zhong W. (2016). Nanofibres for Medical Textiles. Advances in Smart Medical Textiles: Treatments and Health Monitoring.

[56] Zheng Y. (2019). Fabrication on bioinspired surfaces. Bioinspired Design of Materials Surfaces.

[57] Singh R., Eitler D., Morelle R., Friedrich R., Dietel B., Alexiou C., Boccaccini A., Liverani L., Cicha I. (2020). Optimization of cell seeding on electrospun PCL-silk fibroin scaffolds. Eur. Polym. J..

[58] Li J., Chen M., Fan X., Zhou H. (2016). Recent advances in bioprinting techniques: Approaches, applications and future prospects. J. Transl. Med..

[59] Mirdamadi E., Tashman J., Shiwarski D., Palchesko R., Feinberg A. (2020). FRESH 3D bioprinting a full-size model of the human heart. ACS Biomater. Sci. Eng..

[60] Lee A., Hudson A., Shiwarski D., Tashman J., Hinton T., Yerneni S., Bliley J., Campbell P., Feinberg A. (2019). 3D bioprinting of collagen to rebuild components of the human heart. Science.

[61] Senior J., Cooke M., Grover L., Smith A., Senior J., Smith A., Cooke M., Grover L. (2019). Fabrication of Complex Hydrogel Structures Using Suspended Layer Additive Manufacturing (SLAM). Adv. Funct. Mater..

[62] Skylar-Scott M., Uzel S., Nam L., Ahrens J., Truby R., Damaraju S., Lewis J. (2019). Biomanufacturing of organ-specific tissues with high cellular density and embedded vascular channels. Sci. Adv..

[63] Burdis R., Kelly D. (2021). Biofabrication and bioprinting using cellular aggregates, microtissues and organoids for the engineering of musculoskeletal tissues. Acta Biomater..

[64] Ong C., Fukunishi T., Zhang H., Huang C., Nashed A., Blazeski A., Disilvestre D., Vricella L., Conte J., Tung L. (2017). Biomaterial-Free Three-Dimensional Bioprinting of Cardiac Tissue using Human Induced Pluripotent Stem Cell Derived Cardiomyocytes. Sci. Rep..

[65] Noor N., Shapira A., Edri R., Gal I., Wertheim L., Dvir T. (2019). 3D Printing of Personalized Thick and Perfusable Cardiac Patches and Hearts. Adv. Sci..

[66] Loai S., Kingston B.R., Wang Z., Philpott D.N., Tao M., Cheng H.-L.M. (2019). Clinical Perspectives on 3D Bioprinting Paradigms for Regenerative Medicine. Regen. Med. Front..

[67] Murphy S.V., Atala A. (2014). 3D bioprinting of tissues and organs. Nat. Biotechnol..

[68] Aziz A., Geng C., Fu M., Yu X., Qin K., Liu B. (2017). The role of microfluidics for organ on chip simulations. Bioengineering.

[69] Zhao Y., Rafatian N., Feric N., Cox B., Aschar-Sobbi R., Wang E., Aggarwal P., Zhang B., Conant G., Ronaldson-Bouchard K. (2019). A Platform for Generation of Chamber-Specific Cardiac Tissues and Disease Modeling. Cell.

[70] Parsa H., Wang B., Vunjak-Novakovic G. (2017). A microfluidic platform for the high-throughput study of pathological cardiac hypertrophy. Lab Chip.

[71] Zhao Y., Rafatian N., Wang E., Feric N., Lai B., Knee-Walden E., Backx P., Radisic M. (2019). Engineering microenvironment for human cardiac tissue assembly in heart-on-a-chip platform. Matrix Biol..

[72] Jang K., Suh K. (2010). A multi-layer microfluidic device for efficient culture and analysis of renal tubular cells. Lab Chip.

[73] Kilic O., Pamies D., Lavell E., Schiapparelli P., Feng Y., Hartung T., Bal-Price A., Hogberg H., Quinones-Hinojosa A., Guerrero-Cazares H.A. (2016). Levchenko, Brain-on-a-chip model enables analysis of human neuronal differentiation and chemotaxis. Lab Chip.

[74] Grigoryan B., Paulsen S., Corbett D., Sazer D., Fortin C., Zaita A., Greenfield P., Calafat N., Gounley J., Ta A. (2019). Multivascular networks and functional intravascular topologies within biocompatible hydrogels. Am. Assoc. Adv. Sci..

[75] Sontheimer-Phelps A., Chou D., Tovaglieri A., Ferrante T., Duckworth T., Fadel C., Frismantas V., Sutherland A., Jalili-Firoozinezhad S., Kasendra M. (2020). Human Colon-on-a-Chip Enables Continuous In Vitro Analysis of Colon Mucus Layer Accumulation and Physiology. Cell. Mol. Gastroenterol. Hepatol..

[76] Bhise N., Manoharan V., Massa S., Tamayol A., Ghaderi M., Miscuglio M., Lang Q., Zhang Y., Shin S., Calzone G. (2016). A liver-on-a-chip platform with bioprinted hepatic spheroids. Biofabrication.

[77] Seo J., Byun W., Alisafaei F., Georgescu A., Yi Y., Massaro-Giordano M., Shenoy V., Lee V., Bunya V., Huh D. (2019). Multiscale reverse engineering of the human ocular surface. Nat. Med..

[78] Costa P., Albers H., Linssen J., Middelkamp H., Van Der Hout L., Passier R., Van Den Berg A., Malda J., Van Der Meer A. (2017). Mimicking arterial thrombosis in a 3D-printed microfluidic: In vitro vascular model based on computed tomography angiography data. Lab Chip.

[79] Lan D., Shang Y., Su H., Liang M., Liu Y., Li H., Feng Q., Cao X., Dong H. (2021). Facile Fabrication of Hollow Hydrogel Microfiber via 3D Printing-Assisted Microfluidics and Its Application as a Biomimetic Blood Capillary. ACS Biomater. Sci. Eng..

[80] Hong S., Song J. (2022). 3D bioprinted drug-resistant breast cancer spheroids for quantitative in situ evaluation of drug resistance. Acta Biomater..

[81] van Meer B., de Vries H., Firth K., van Weerd J., Tertoolen L., Karperien H., Jonkheijm P., Denning C., IJzerman A., Mummery C. (2017). Small molecule absorption by PDMS in the context of drug response bioassays. Biochem. Biophys. Res. Commun..

[82] Campbell S., Wu Q., Yazbeck J., Liu C., Okhovatian S., Radisic M. (2020). Beyond polydimethylsiloxane: Alternative materials for fabrication of organ on a chip devices and microphysiological systems. ACS Biomater. Sci. Eng..

[83] Abaci H., Shuler M. (2015). Human-on-a-chip design strategies and principles for physiologically based pharmacokinetics/pharmacodynamics modeling. Integr. Biol..

[84] Kim Y., Ko H., Kwon I., Shin K. (2016). Extracellular matrix revisited: Roles in tissue engineering. Int. Neurourol. J..

[85] NIH Stem Cell, NIH Stem Cell Information Home Page—Stem Cell Basics, In Stem Cell Information. https://stemcells.nih.gov/.

[86] Doss M., Sachinidis A. (2019). Current Challenges of iPSC-Based Disease Modeling and Therapeutic Implications. Cells.

[87] Ricklefs M., Korossis S., Haverich A., Schilling T. (2017). Polymeric Scaffolds for Bioartificial Cardiovascular Prostheses. Scaffolds in Tissue Engineering—Materials, Technologies and Clinical Applications.

[88] Wang X., Ao Q., Tian X., Fan J., Tong H., Hou W., Bai S. (2017). Gelatin-based hydrogels for organ 3D bioprinting. Polymers.

[89] Xu B., Li Y., Deng B., Liu X., Wang L., Zhu Q. (2017). Chitosan hydrogel improves mesenchymal stem cell transplant survival and cardiac function following myocardial infarction in rats. Exp. Ther. Med..

[90] Wang Z., Lee S., Cheng H., Yoo J., Atala A. (2018). 3D bioprinted functional and contractile cardiac tissue constructs. Acta Biomater..

[91] Gaetani R., Doevendans P., Metz C., Alblas J., Messina E., Giacomello A., Sluijter J. (2012). Cardiac tissue engineering using tissue printing technology and human cardiac progenitor cells. Biomaterials.

[92] Maiullari F., Costantini M., Milan M., Pace V., Chirivì M., Maiullari S., Rainer A., Baci D., Marei H., Seliktar D. (2018). A multi-cellular 3D bioprinting approach for vascularized heart tissue engineering based on HUVECs and iPSC-derived cardiomyocytes. Sci. Rep..

[93] Mehrali M., Thakur A., Kadumudi F., Pierchala M., Cordova J., Shahbazi M., Mehrali M., Pennisi C., Orive G., Gaharwar A. (2019). Pectin Methacrylate (PEMA) and Gelatin-Based Hydrogels for Cell Delivery: Converting Waste Materials into Biomaterials. ACS Appl. Mater. Interfaces.

[94] Kc P., Hong Y., Zhang G. (2019). Cardiac tissue-derived extracellular matrix scaffolds for myocardial repair: Advantages and challenges. Regen. Biomater..

[95] Dong D., Li J., Cui M., Wang J., Zhou Y., Luo L., Wei Y., Ye L., Sun H., Yao F. (2016). In Situ “clickable” Zwitterionic Starch-Based Hydrogel for 3D Cell Encapsulation. ACS Appl. Mater. Interfaces.

[96] Reys L., Silva S., Da Costa D.S., Oliveira N., Mano J., Reis R., Silva T. (2016). Fucoidan Hydrogels Photo-Cross-Linked with Visible Radiation As Matrices for Cell Culture. ACS Biomater. Sci. Eng..

[97] Chen Q., Zhu C., Thouas G.A. (2012). Progress and challenges in biomaterials used for bone tissue engineering: Bioactive glasses and elastomeric composites. Prog. Biomater..

[98] Family R., Solati-Hashjin M., Nik S., Nemati A. (2012). Surface modification for titanium implants by hydroxyapatite nanocomposite. Casp. J. Intern. Med..

[99] Lee H., Byun S., Cho S., Yang B. (2019). Past, present, and future of regeneration therapy in oral and periodontal tissue: A review. Appl. Sci..

[100] Ho C., Mishra A., Lin P., Ng S., Yeong W., Kim Y., Yoon Y. (2017). 3D Printed Polycaprolactone Carbon Nanotube Composite Scaffolds for Cardiac Tissue Engineering. Macromol. Biosci..

[101] Mironov A.V., Grigoryev A., Krotova L., Skaletsky N., Popov V., Sevastianov V. (2017). 3D printing of PLGA scaffolds for tissue engineering. J. Biomed. Mater. Res. A.

[102] Savoji H., Huyer L.D., Mohammadi M., Lai B.L., Rafatian N., Bannerman D., Shoaib M., Bobicki E., Ramachandran A., Radisic M. (2020). 3D Printing of Vascular Tubes Using Bioelastomer Prepolymers by Freeform Reversible Embedding. ACS Biomater. Sci. Eng..

[103] Ulery B., Nair L., Laurencin C. (2011). Biomedical applications of biodegradable polymers. J. Polym. Sci. Part B Polym. Phys..

[104] Kunduru K., Basu A., Domb A. (2016). Biodegradable Polymers: Medical Applications. Encyclopedia of Polymer Science and Technology.

[105] Kasper F., Tanahashi K., Fisher J., Mikos A. (2009). Synthesis of poly(propylene fumarate). Nat. Protoc..

[106] Kinard L., Kasper F., Mikos A. (2012). Synthesis of oligo(Poly(ethylene glycol) fumarate). Nat. Protoc..

[107] Bahraminasab M., Sahari B., Edwards K., Farahmand F., Arumugam M. (2012). Aseptic loosening of femoral components—Materials engineering and design considerations. Mater. Des..

[108] Ma X., Liu J., Zhu W., Tang M., Lawrence N., Yu C., Gou M., Chen S. (2018). 3D bioprinting of functional tissue models for personalized drug screening and in vitro disease modeling. Adv. Drug Deliv. Rev..

[109] Kengla C., Kidiyoor A., Murphy S.V. (2017). Bioprinting Complex 3D Tissue and Organs. Kidney Transplantation, Bioengineering, and Regeneration: Kidney Transplantation in the Regenerative Medicine Era.

[110] Skardal A. (2015). Bioprinting essentials of cell and protein viability. Essentials of 3D Biofabrication and Translation.

[111] Salaris F.A. (2019). Rosa, Construction of 3D in vitro models by bioprinting human pluripotent stem cells: Challenges and opportunities. Brain Res..

[112] Augustine R., Kalva S., Ahmad R., Zahid A., Hasan S., Nayeem A., McClements L., Hasan A. (2021). 3D Bioprinted cancer models: Revolutionizing personalized cancer therapy. Transl. Oncol..

[113] Chen C., Sereti K., Wu B., Ardehali R. (2015). Translational aspects of cardiac cell therapy. J. Cell. Mol. Med..

[114] Liao S.-Y., Tse H.-F. (2013). Multipotent (adult) and pluripotent stem cells for heart regeneration: What are the pros and cons?. Stem Cell Res. Ther..

[115] Gálvez-Montón C., Prat-Vidal C., Roura S., Soler-Botija C., Bayes-Genis A. (2013). Cardiac Tissue Engineering and the Bioartificial Heart. Rev. Española De Cardiol. (Engl. Ed.).

[116] Dmitriev R., Borisov S., Düssmann H., Sun S., Müller B., Prehn J., Baklaushev V., Klimant I., Papkovsky D. (2015). Versatile conjugated polymer nanoparticles for high-resolution O_2_ imaging in cells and 3D tissue models. ACS Nano.

[117] Müller B., Zhdanov A.V., Borisov S., Foley T., Okkelman I., Tsytsarev V., Tang Q., Erzurumlu R., Chen Y., Zhang H. (2018). Nanoparticle-Based Fluoroionophore for Analysis of Potassium Ion Dynamics in 3D Tissue Models and In Vivo. Adv. Funct. Mater..

[118] Goliwas K., Richter J., Pruitt H., Araysi L., Anderson N., Samant R., Lobo-Ruppert S., Berry J., Frost A. (2017). Methods to Evaluate Cell Growth, Viability, and Response to Treatment in a Tissue Engineered Breast Cancer Model. Sci. Rep..

[119] Bardsley K., Deegan A., El Haj A., Yang Y. (2017). Current state-of-the-art 3D tissue models and their compatibility with live cell imaging. Adv. Exp. Med. Biol..

[120] Elliott A. (2020). Confocal Microscopy: Principles and Modern Practices. Curr. Protoc. Cytom..

[121] Fischer E., Hansen B., Nair V., Hoyt F., Dorward D. (2012). Scanning electron microscopy. Curr. Protoc. Microbiol..

[122] Misof B., Roschger P., Fratzl P. (2011). Imaging mineralized tissues in vertebrates. Comprehensive Biomaterials.

[123] Ishikawa-Ankerhold H., Ankerhold R., Drummen G. (2012). Advanced fluorescence microscopy techniques-FRAP, FLIP, FLAP, FRET and FLIM. Molecules.

[124] Donius A., Bougoin S.V., Taboas J. (2016). FRET imaging in three-dimensional hydrogels. J. Vis. Exp..

[125] Sekar R., Periasamy A. (2003). Fluorescence resonance energy transfer (FRET) microscopy imaging of live cell protein localizations. J. Cell Biol..

[126] Fujimoto J., Pitris C., Boppart S., Brezinski M. (2000). Optical coherence tomography: An emerging technology for biomedical imaging and optical biopsy. Neoplasia.

[127] Wang L., Xu M., Zhang L., Zhou Q., Luo L. (2016). Automated quantitative assessment of three-dimensional bioprinted hydrogel scaffolds using optical coherence tomography. Biomed. Opt. Express.

[128] Wang S., Larina I.V. (2017). High-resolution imaging techniques in tissue engineering. Monitoring and Evaluation of Biomaterials and Their Performance In Vivo.

[129] Meddens M., de Keijzer S., Cambi A. (2014). High Spatiotemporal Bioimaging Techniques to Study the Plasma Membrane Nanoscale Organization. Fluorescence Microscopy: Super-Resolution and Other Novel Techniques.

[130] Lee S., Lee B., Lee J., Kim S., Kim J., Jeong Y., Jin S. (2013). Microscale diffusion measurements and simulation of a scaffold with a permeable strut. Int. J. Mol. Sci..

[131] Zheng K., Rupnick M., Liu B., Brezinski M. (2009). Three Dimensional OCT in the Engineering of Tissue Constructs: A Potentially Powerful Tool for Assessing Optimal Scaffold Structure. Open Tissue Eng. Regen. Med. J..

[132] Wang L., Xu M., Luo L., Zhou Y., Si P. (2018). Iterative feedback bio-printing-derived cell-laden hydrogel scaffolds with optimal geometrical fidelity and cellular controllability. Sci. Rep..

